# Discovery of a Bradykinin B2 Partial Agonist Profile of Raloxifene in a Drug Repurposing Campaign

**DOI:** 10.3390/ijms22010257

**Published:** 2020-12-29

**Authors:** Patricia Gomez-Gutierrez, Juan J. Perez

**Affiliations:** Department of Chemical Engineering, Universitat Politecnica de Catalunya, ETSEIB, Av. Diagonal, 647, 08028 Barcelona, Spain; ondopasa@gmail.com

**Keywords:** nonpeptide bradykinin partial agonist, drug repurposing, Covid-19 therapies, raloxifene

## Abstract

Covid-19 urges a deeper understanding of the underlying molecular mechanisms involved in illness progression to provide a prompt therapeutical response with an adequate use of available drugs, including drug repurposing. Recently, it was suggested that a dysregulated bradykinin signaling can trigger the cytokine storm observed in patients with severe Covid-19. In the scope of a drug repurposing campaign undertaken to identify bradykinin antagonists, raloxifene was identified as prospective compound in a virtual screening process. The pharmacodynamics profile of raloxifene towards bradykinin receptors is reported in the present work, showing a weak selective partial agonist profile at the B2 receptor. In view of this new profile, its possible use as a therapeutical agent for the treatment of severe Covid-19 is discussed.

## 1. Introduction

About 20% of patients infected with SARS-Cov-2 develop respiratory complications, including pneumonia, that may evolve to severe hypoxemia, acute respiratory distress syndrome (ARDS), pulmonary edema, shock, metabolic acidosis, coagulation dysfunction and multiple organ functional failure [1,2]. Covid-19 patients with severe symptoms exhibit an immune response characterized by elevated serum levels of diverse proinflammatory molecules, including interleukin-6 (IL-6) [3,4,5,6]. Presently, these patients are treated with anti-inflammatory drugs like dexamethasone [7] or cytokine inhibitors, combined with antibiotics to treat secondary infections, sometimes combined with remdesivir, an antiviral originally developed to treat hepatitis C and tested against the Ebola virus disease to reduce the viral load [8].

Treatment of Covid-19 urges a deeper understanding of the underlying molecular mechanisms involved in disease progression to provide a prompt therapeutical response with an adequate use of available drugs, including drug repurposing. Indeed, there is a number of ongoing clinical trials to test the efficacy of diverse FDA-approved drugs against Covid-19. Among them, a few involve assessment of the therapeutic efficacy of IL-6 inhibition, unfortunately without reporting conclusive results [9,10,11]. Recently, it was proposed that dysregulated bradykinin (BK) signaling could be the trigger of the cytokine storm observed in patients with severe Covid-19 [12,13,14,15]. The rational for this hypothesis relies on the increased availability of BK and its metabolite des-Arg^9^-BK in Covid-19 patients, due to the downregulation of angiotensin converting enzyme 2 (ACE2), the entry protein of SARS-Cov-2 [16]. Indeed, ACE2 downregulation increases the amount of angiotensin I processed by the angiotensin-converting enzyme (ACE) with a concomitant decrease of its degradation activity on BK. Moreover, levels of des-Arg^9^-BK are also increased due to the diminished degradative activity of ACE2 caused by its downregulation [12]. The BK hypothesis is consistent with the observed elevated levels of IL-6 in Covid-19 patients [17]. This new perspective suggests that inhibition of BK signaling may be a suitable therapy to avoid the cytokine storm and its consequences. Indeed, as a proof of principle, preliminary results from a limited study suggests that inhibition of the BK signaling is associated with a decrease of the oxygen supplementation needed by Covid-19 patients [18].

Nowadays, icatibant is the only BK antagonist approved as therapeutic agent for the treatment of hereditary angioedema [19], in spite of the number of selective BK ligands described in the literature [20]. The discovery of the bradykinin hypothesis prompted the launch of an ongoing clinical trial to assess the benefits of icatibant for the treatment of covid-19 patients [21]. In other to swiftly test the efficacy of alternative BK antagonists with diverse pharmacological profiles for the treatment of Covid-19, drug repurposing represents a valuable strategy [22]. To this aim, we recently carried out a virtual screening study on the DrugBank database [23] aimed at identifying BK ligands among drugs already approved by the FDA [24]. The study was carried out using four points common to the pharmacophores derived for the B1 and B2 bradykinin receptors recently reported that were used as queries for the search [25,26,27]. Hits identified were subsequently subjected to a docking study using the Molecular Operating Environment (MOE) program [28].

The virtual screening process permitted the identification of a small set of drugs as prospective nonselective BK ligands, as described elsewhere [24]. Specifically, the DrugBank database, containing a total of 1703 molecules, was screened for compounds that fulfilled a common pharmacophore for the B1 and B2 receptors. The study identified eight drugs including raloxifene, sildenafil, cefepime, cefpirome, imatinib, ponatinib, abemaciclib and entrectinib. Subsequently, the eight compounds were purchased and tested for their capacity to displace a reference ligand in any of the two bradykinin receptors B1 and B2, respectively at 20 µM. Three of the compounds including raloxifene, sildenafil and cefepime displaced the reference ligand from B2, which represents a 40% success rate as found in similar studies [29]. However, none of the compounds exhibited affinity for the B1 receptor at this concentration. This might be due to the steric hindrance that was not properly included in the virtual screening search. Raloxifene, the most potent antagonist identified was further investigated in a functional study in vitro, exhibiting an IC_50_ of ~16 µM. Moreover, the compound showed a weak partial agonist profile with a maximal activity of ~20%. Ralixofene is a selective estrogen receptor modulator, exerting estrogen agonist action in some target tissues while acting as an estrogen antagonist in others [30]. The compound was approved a few years ago for the treatment of osteoporosis in postmenopausal women, as well as for cancer chemoprotection. The discovery of a new pharmacodynamic profile of relixofene as a BK partial agonist opens an opportunity for its repurposing as a therapeutic agent for the treatment of severe Covid-19.

## 2. Results and Discussion

In order to have a preliminary insight into the affinity of ralixofene towards the bradykinin receptors, the compound was first tested at 20 µM for its capacity to displace a reference ligand from B1 and B2, respectively. Specifically, desArg^10^-kallidin was used in the case of the B1 receptor and NPC 567 for the B2 receptor. The results of this study indicate that at this concentration, ralixofene does not displace desArg^10^-kallidin at the B1 receptor, whereas the reference ligand at the B2 receptor is displaced ~54%. We subsequently proceeded to carry out a study of the pharmacodynamic profile of the compound towards the BK B2 receptor in vitro, using a functional efficacy measurement [31]. Accordingly, we tested its capacity to antagonize BK on Chinese Hamster Ovary (CHO) cells stably transfected with the B2 receptor.

**Figure 1:**
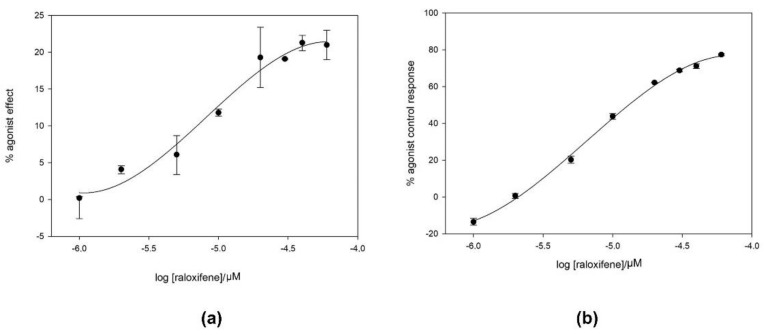
Functional efficacy study of raloxifene on CHO cells stably transfected with the B2 receptor (N = 2). (**a**) Study of the agonistic effect of the compound compared to bradykinin; (**b**) study of the efficacy of the compound antagonizing bradykinin.

As can be seen in Figure 1a, raloxifene antagonizes BK with an IC_50_ ~16 µM with an apparent dissociation constant K_B_ = 1.8 µM, computed using the modified Cheng Prusoff equation [32]:

$$K_{B}=\frac{{\mathrm{IC}}_{50}}{1+\frac{A}{{\mathrm{EC}}_{50}}}$$

where, IC_50_ = 16 µM corresponds to raloxifene and EC_50_ = 2.4 pM corresponds to BK, and A = 0.03 nM is the concentration of BK that was used in the experiments. We also tested its activity as an agonist on CHO cells stably transfected with the B2 receptor. Present results also showed (Figure 1b) that the compound exhibited a partial agonistic profile, with a maximum effect of 20% of BK that was achieved at around 20 µM.

**Figure 2:**
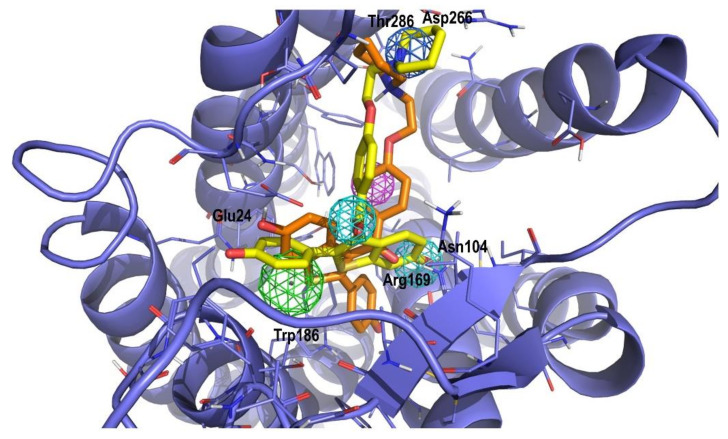
Superposition of the prospective bound conformation of raloxifene onto the B2 receptor (orange) and the matching structure of raloxifene (yellow) from the virtual screening process. The 3D structure of the B2 receptor was constructed by homology modeling and reported previously [27]. Color spheres represent the pharmacophore points characteristic of the ligands binding to B2 receptor: dark blue represents a positive charge moiety; magenta a hydrogen bond accepting center; light blue a hydrogen bond donor/acceptor center; green an aromatic/lipophilic center.

The antagonistic profile of the compound can be rationalized from docking studies. Figure 2 shows the prospective bound conformation of raloxifene (orange) to the B2 receptor, superimposed with the conformation matching the query (yellow) from the virtual screening process. Despite fulfilling the four pharmacophore points, the directionality of the hydrogen bond interactions (not included in the query) was not optimal. However, in the docking process, the molecule was relaxed to satisfy new interactions that may improve its docking score. As can be seen in Figure 2, from its initial position (yellow), the molecule tilts to get a few favorable interactions (orange). Specifically, the phenoxyl moiety sacrifices a hydrogen bond interaction with Arg^169^ that is compensated with a new one with Asn^104^. This in turn, permits the phenoxyl aromatic ring to get closer to Trp^86^ and the carbonyl group establishes a hydrogen bond interaction with Arg^169^. On the other hand, some interactions are weakened, for example, the interaction of the bicycle moiety with Trp^86^ and the interaction of the pyridine nitrogen with Asp^266^. Partial fulfilment of the pharmacophore may explain the low IC_50_ exhibited by raloxifene [26]. On the other hand, the fact that raloxifene does not bind to the B1 can only be explained by steric interactions, since most of the residues between the two receptors are conserved [27]. Inspection of the results of the docking study reveals that the pyridine nitrogen of raloxifene exhibits a hydrogen bond with Thr^286^ that cannot not be established in B1, since the position is occupied by Leu^294^.

Bradykinin signaling though the B2 receptor induces a proinflammatory action as well as a hypotensive effect [33]. The proinflammatory activity of BK involves the stimulation of cytokines, like IL-6 and IL-8, through the MAPK/AP-1 signaling axis [17,34], whereas the hypotensive effects are due to vasodilation and increased vascular permeability. Due to the partial agonist profile of raloxifene, it is expected that the compound antagonizes the proinflammatory effects of BK, modulating the levels of diverse cytokines, including IL-6. To support this hypothesis, in clinical trials aimed at understanding the effect of hormone replacement therapy for menopausal women on the risk of cardiovascular disease, in contrast to other compounds raloxifene did not exhibit a proinflammatory profile [35,36] that could, in part, be explained by its antagonistic effect on the BK B2 receptor.

The beneficial vascular effects of ralixofene could, in part, be explained by its partial agonist profile towards the BK B2 receptor [36]. Thus, in a study aimed at assessing the vasoprotective effects of the compound, rats treated with raloxifene showed an increased reduction of systolic blood pressure on administration of bradykinin, suggesting an enhanced bioavailability of NO in these animals [37]. Moreover, diverse experiments subsequently confirmed a role of raloxifene inducing endothelium-dependent relaxation as due to the upregulation of nitric-oxide synthase (eNOS) expression [38,39,40]. Although these results can be due to the partial agonist profile of raloxifene reported in this work [33], the effect can also be explained as due to the activation of the GPR30 receptor [41]. Accordingly, further experimental work is necessary to differentiate both effects.

Present results strongly support the need to perform a deeper investigation on the use of raloxifene as a therapeutic agent for the treatment of Covid-19. As shown in the present communication, its pharmacological profile as a partial BK B2 agonist is expected to antagonize the inflammatory action of BK, shown to be upregulated in Covid-19 patients [12,13,14,15]. This, in turn, will produce a reduction of cytokine levels, including IL-6, which is the primary proinflammatory cytokine associated with the severity of the illness [5,6]. On the other hand, its partial agonism profile is expected to maintain a base level of BK, preserving its beneficial cardiovascular effects [42]. Despite the novel beneficial effects identified, caution should be paid due to the necessary higher dose required for the compound to be used for a novel therapeutic use. The treatment of osteoporosis requires an oral dose between 30 to 150 mg/d. Taking into account its poor bioavailability of 2%, a single dose of 100 mg gives a maximal concentration in plasma of ~2 nM [43]. Concentrations necessary for the therapeutical benefit of raloxifene towards the BK B2 receptor are expected to be, at most, around 100 times higher, which is within the therapeutic window of the drug [44].

The results of this work complement recent comments suggesting the use of raloxifene for the treatment of Covid-19 [45], based on its capacity to inhibit the IL-6/STAT3 signaling pathway [46,47]. In addition, the presumed antiviral profile of ralixofene against the SARS-Cov2 virus through its capability to block the two-pore channel TCP2 [48], as found for the ebola virus [49] has been underlined. In conclusion, the pharmacodynamic profile exhibited by ralidoxene described in the present report represents a reasonable base to test the compound for the treatment of Covid-19. Very recently a clinical trial in Italy has been approved to test ralixofene in COVID-19 patients with mild symptoms [50].

## 3. Materials and Methods

### 3.1. Computational Methods

Vitual screening on the DrugBank [23] was carried out using the Molecular Operating Environment (MOE) program [28], using four common points of the BK B1 and B2 pharmacophores described previously [25,26] that were used as a query. Previously, a 3D DrugBank database containing the 3D structure, together with a set of conformations of each molecule, was constructed as explained elsewhere [24]. Virtual screening was carried out on the subset of molecules with molecular weight between 200 and 600 (a total of 1703 molecules). Hits identified in the screening process were docked in the 3D models of the B1 and B2 bradykinin receptors, using a set of unique conformations resulting from thorough conformational searches for the diverse ligands studied and rank ordered by means of the MOE program [28].

### 3.2. In Vitro Assays

#### 3.2.1. Ligand Displacement Assays

Raloxifene was tested for its ability to displace reference ligands on human recombinant bradykinin B1 or B2 receptors, respectively, expressed in CHO cells at 20 µM. For this purpose, first saturation isotherms were obtained with reference ligands ([^3^H] des-Arg^10^-BK (0.35 nM) in the case of B1, and [^3^H]-bradykinin (0.2 nM) for B2) incubated for 60 min at room temperature. Nonspecific binding was evaluated by adding a reference compound (desArg^9^[Leu^8^]-BK at 10 µM in the case of B1 and BK at 1 µM for B2). Antagonism of raloxifene was measured as a percent inhibition of specific binding of [^3^H]-bradykinin as control, obtained in the presence of raloxifene at 20 µM and using as reference compounds desArg^10^-KD for B1 and NPC-567 for B2.

#### 3.2.2. Functional Efficacy Assays

Measurementent of the efficacy of the compound on the B2 receptor was carried out following the protocol described elsewhere [31]. The method is based on measuring differences in intracellular Ca^2+^ concentrations produced in diverse conditions on CHO cells expressing recombinant human B2 receptor by fluorometry. Agonism was measured through the capacity of the compound to increase Ca^2+^ concentration compared to BK (EC_50_ ~ 2.4 pM). Antagonism was measured through the capacity to antagonize BK based on the reduction of Ca^2+^ concentration.

## 4. Conclusions

The aim of the present study was to characterize the pharmacological profile of raloxifene towards the bradykinin receptors. The compound was identified as a micromolar ligand of the B2 receptor by virtual screening in a drug repurposing campaign. Results showed that raloxifene is a weak partial agonist toward the B2 receptor, with a 19% efficacy compared to bradykinin, with an apparent dissociation constant K_B_ ~ 21 µM. The discovery of the bradykinin pharmacodynamics profile of raloxidene explains in part its observed vascular beneficial effects, although they could also be due to activation of the GPR30 receptor signaling pathway. On the other hand, it acts as a bradykinin antagonist for B2 with an IC_50_ ~ 21 µM.

Based on the bradykinin hypothesis on the trigger of the cytokine storm in severe Covid-19 [12,13,14,15], these results suggest that the compound could be used for its treatment. Present results support previous claims suggesting the use of raloxifene based on its capacity to inhibit the IL-6/STAT3 signaling pathway [45], and also for being a prospective antiviral agent for SARS-Cov2 as inhibitor of the two-pore channel TCP2 [48]. Very recently a clinical trial in Italy has been approved to test its efficacy in COVID-19 patients with mild symptoms [50].

## Data Availability

The data presented in this study are available upon request from the corresponding author.
